# Supplementary figures and images for: Discovering the fish fauna of a lagoon from the southeast of the Yucatan Peninsula, Mexico, using DNA barcodes
Source: PeerJ. 2023 Nov 2;11:e16285. doi: 10.7717/peerj.16285 (PMC10625761; doi:10.7717/peerj.16285)

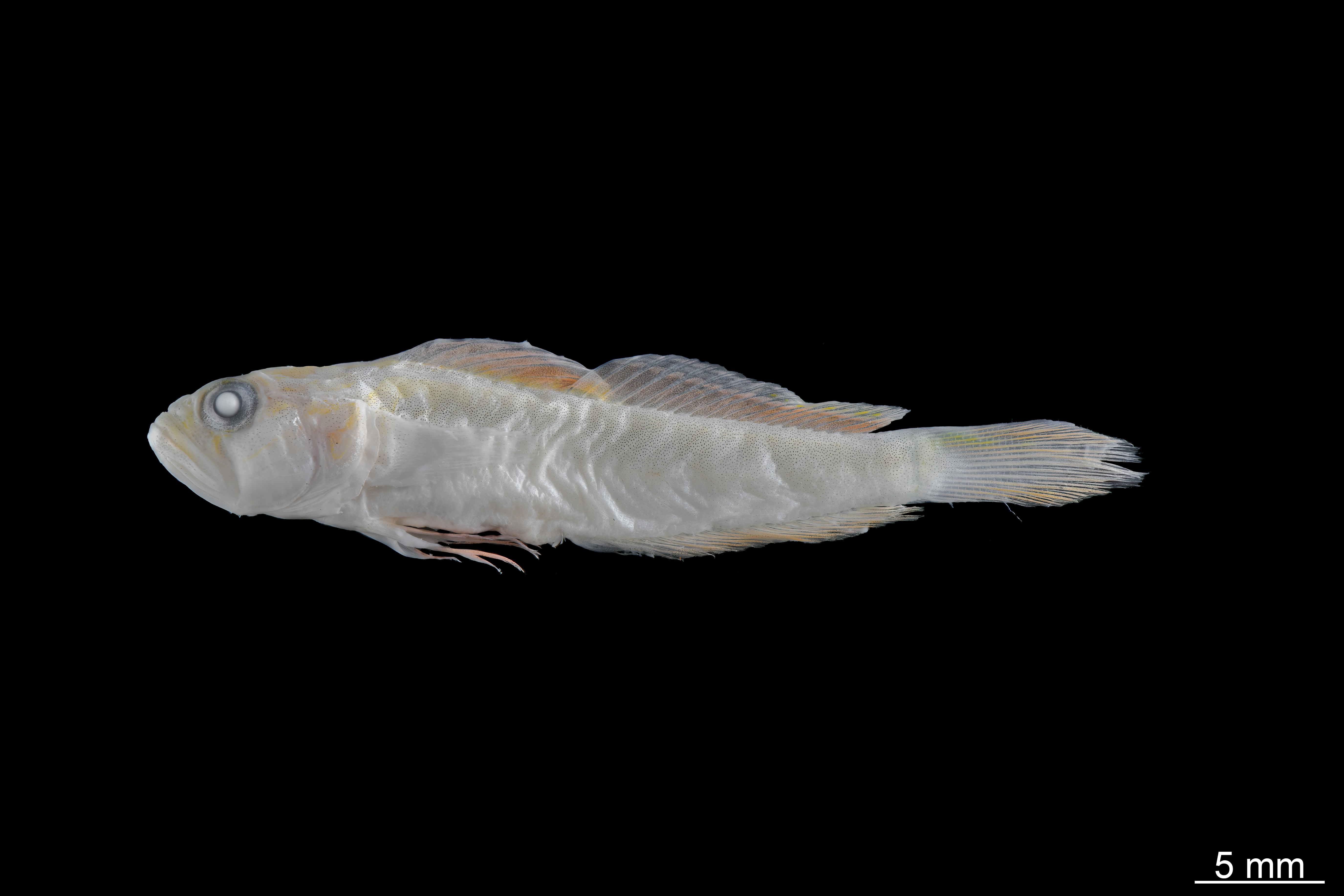

Supplement: Supplemental Information 3 — A) Pregnant female B) male. [file peerj-11-16285-s003.jpg]
